# Human protein C concentrate in the treatment of purpura fulminans: a retrospective analysis of safety and outcome in 94 pediatric patients

**DOI:** 10.1186/cc9226

**Published:** 2010-08-19

**Authors:** Alex Veldman, Doris Fischer, Flora Y Wong, Wolfhart Kreuz, Michael Sasse, Bruno Eberspächer, Ulrich Mansmann, Rudolf Schosser

**Affiliations:** 1 Monash Newborn, Monash Medical Centre; The Ritchie Centre, Monash Institute for Medical Research and Department of Pediatrics, Monash University, 246 Clayton RD, Clayton 3168, Melbourne, Australia; 2 Department of Pediatrics, J.W. Goethe University Hospital, Theodor Stern Kai 7, 60590 Frankfurt/Main, Germany; 3 Department of Pediatric Cardiology and Pediatric Intensive Care, University Childrens Hospital Hannover, Carl Neuberg Str. 1, 30625 Hannover, Germany; 4 Baxter BioScience, EdisonStr. 4, 85716 Unterschleißheim, Germany; 5 Department of Medical Informatics, Biometry, and Epidemiology, L. Maximilian University, Marchioninistr. 15, 81377 Munich, Germany

## Abstract

**Introduction:**

Purpura fulminans (PF) is a devastating complication of uncontrolled systemic inflammation, associated with high incidence of amputations, skin grafts and death. In this study, we aimed to clarify the clinical profile of pediatric patients with PF who improved with protein C (PC) treatment, explore treatment effects and safety, and to refine the prognostic significance of protein C plasma levels.

**Methods:**

In Germany, patients receiving protein C concentrate (Ceprotin^®^, Baxter AG, Vienna, Austria) are registered. The database was used to locate all pediatric patients with PF treated with PC from 2002 to 2005 for this national, retrospective, multi-centered study.

**Results:**

Complete datasets were acquired in 94 patients, treated in 46 centers with human, non-activated protein C concentrate for purpura fulminans. PC was given for 2 days (median, range 1-24 days) with a median daily dose of 100 IU/kg. Plasma protein C levels increased from a median of 27% to a median of 71% under treatment. 22.3% of patients died, 77.7% survived to discharge. Skin grafts were required in 9.6%, amputations in 5.3%. PF recovered or improved in 79.8%, remained unchanged in 13.8% and deteriorated in 6.4%. Four adverse events occurred in 3 patients, none classified as severe. Non-survivors had lower protein C plasma levels (*P *< 0.05) and higher prevalence of coagulopathy at admission (*P *< 0.01). Time between admission and start of PC substitution was longer in patients who died compared to survivors (*P *= 0.03).

**Conclusions:**

This retrospective dataset shows that, compared to historic controls, only few pediatric patients with PF under PC substitution needed dermatoplasty and/or amputations. Apart from epistaxis, no bleeding was observed. Although the data comes from a retrospective study, the evidence we present suggests that PC had a beneficial impact on the need for dermatoplasty and amputations, pointing to the potential value of carrying out a prospective randomised controlled trial.

## Introduction

Dermal and systemic thrombosis of the microcirculation, referred to as purpura fulminans (PF), is a devastating complication of widespread endothelial destruction due to uncontrolled systemic inflammation, associated with a high incidence of multiple organ failure, need for amputations, skin grafts and death. PF most frequently occurs in the pediatric age group, with a peak incidence in infants (1 to 3 years of age) and adolescents (16 to 18 years of age). Although most frequently seen in the context of severe septic shock, in particular in patients with meningococcemia, PF also occurs in the rare scenario of homozygous or double heterozygous protein C (PC) deficiency [1]. Indeed, the clinical manifestation of a severe PC deficiency in the form of PF has resulted in the description of PF as the clinical symptom of an acute PC pathway failure [2]. Consequently, many intensivists have used PC substitution in patients with PF, mostly with promising results [3-6]. The biological rationale for this use was the anti-coagulant, anti-inflammatory, pro-fibrinolytic, anti-apoptotic and barrier enhancing action of PC [7-10].

This retrospective multi-centered study analyzes clinical features, safety and outcome in 94 pediatric and adolescent patients with PF who received a human non-activated PC concentrate as rescue therapy in Germany from 2002 to 2005. In this study, we aimed to clarify the clinical profile of pediatric patients with PF who improved with PC treatment, explore treatment effects and safety, and to refine the prognostic significance of PC plasma levels. Finally, this study will help to establish hypotheses and endpoints for future prospective studies on human PC in patients with PF.

## Materials and methods

**Figure 1:**
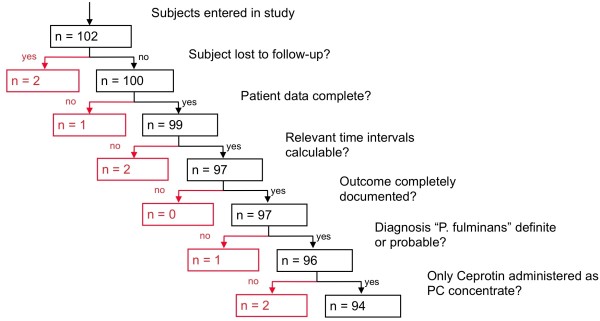
**Flow of patients through the study and exclusions of patients**. The diagnosis of purpura fulminans (PF) was regarded as definite in the presence of livid to partly necrotic lesions of irregular shape and with sharp, clearly defined borders with either rapid progression or already ubiquitous appearance. The diagnosis of PF was regarded as probable in the presence of livid to partly necrotic lesions of irregular shape and with sharp, clearly defined borders. The diagnosis of PF was regarded as unclear in the presence of just livid to partly necrotic lesions without any of the other criteria. The diagnosis of PF was not confirmed in any patient with lesions not fulfilling the above defined criteria. PC, protein C.

The ethics committee of the J.W. Goethe University, Frankfurt, Germany, approved the study protocol for this retrospective, multi-center study. Patients who received a human plasma-derived, virus-inactivated PC concentrate (Ceprotin^®, ^Baxter AG, Vienna, Austria) in Germany were registered by the manufacturer due to a post-marketing commitment requested by the European Medicines Agency. This German database was used to locate all pediatric patients treated with PC concentrate from 2002 to 2005. The flow of the patients throughout the study is displayed in Figure 1. If a patient who received PC concentrate was identified, the principal investigator contacted the treating physician with an invitation to participate in the study. If the treating physician agreed to participate, the hospital was visited by a medical monitor (physician) for standardized (form-based clinical reporting) data collection. For analysis, patients were stratified into three outcome groups by survival to hospital discharge and complications (negative outcome = death and/or amputation; intermediate outcome = survival with skin grafts/dermatoplasty; positive outcome = survival without amputations or skin grafts). As data were collected on anonymous forms, the ethics committee waived the need for informed consent by the patient or relatives.

### Statistics

Descriptive statistics were used for categorical (tables, rates, 95% confidence intervals (CI)) and continuous (quartiles, minimum, maximum, mean, standard deviation) variables. Differences in categorical variables between groups were tested by the chi-square test. Differences in continuous variables between groups were tested by the non-parametric Mann-Whitney U test. Comparisons of relevant parameters were performed between survivors and non-survivors. The relevance of PC plasma levels at admission on the probability to survive the disease was assessed by a logistic regression and an odds ratio was calculated to quantify the influence of the level of PC plasma activity at admission on survival to discharge.

## Results

### Demographics

Of the 102 patients located, 94 entered the final analysis (Figure 1): 52 (55.3%) were male and 42 (44.7%) female; 8 (8.9%) were newborn (< 28 days old), 36 (38.3%) were infants (28 days to 2 years), 29 (30.9%) were children (2 to 12 years) and 21 (22.3) were adolescents (12 to 18 years).

Patients were treated in 46 different centers, with 36 centers treating 2 or less patients and 10 centers treating 3 or more patients. None of the centers participated in or recruited pediatric patients for treatment studies with activated PC at the time of this study.

### Origin of PF

PF was the result of acquired sepsis rather than congenital PC deficiency in all patients. *Neisseria meningitides *was isolated in 75 (79.8%) patients, in 52 of those whose sero-groups were specified.

### Survival and outcome of PF

Twenty-one (22.3%) patients died at a mean duration of two days, and 73 (77.7%) patients survived to discharge. There was no significant difference in age or gender between survivors and patients who did not survive. Skin grafts were required in nine (9.6%) and amputations in five (5.3%) patients.

PF recovered or improved in 75 (79.8%) patients, remained unchanged in 13 (13.8%) patients and deteriorated in 6 (6.4%) patients.

### Shock

At admission, no difference in mean arterial pressure was detected between survivors and non-survivors (median 61.5 vs. 70 mmHg, *P *= 0.168). However, already at admission, survivors presented with lower heart rates (median 150 vs. 185 bpm, *P *= 0.0132) higher Glasgow coma scale scores (mean 12.5 vs. 9.9, *P *= 0.027) and less negative base excess compared with non-survivors (median -4.95 vs. -11.85 mmol/l, *P *= 0.021).

A total of 63 patients received inotropic support for a median duration of two days.

### Protein C treatment

Non-survivors had significantly lower PC plasma activity at admission than survivors (median 10 vs. 30%, *P *= 0.011). A higher level of PC plasma activity by 1% at admission improved the odds to survive significantly (*P *= 0.0285) by a factor of 1.06 (95% CI for odds ratio = 1.01 to 1.12). PC was given for a median of 33 hours (range 1 to 645 hours) with a median daily dose of 100 IU/kg (range 28 to 375 IU/kg). PC was administered as a bolus every four to six hours in 78 patients, and as an initial bolus followed by continuous infusion in the remaining 16 patients. There was no significant difference in survival between the bolus group (21.8% died) and the bolus plus infusion group (25.0% died; *P *= 0.961). Plasma PC levels increased from a median of 27% (range 1 to 75%) prior to PC treatment to a median of 71% under treatment (range 14 to 184%, Table 1). Once under PC substitution, there was no significant difference in plasma PC levels between survivors and non-survivors (*P *= 0.605).

**Table 1:** Clinical and laboratory parameters of all patients, survivors, non-survivors before and during treatment with protein C concentrate

	All	Survived (*n *= 73)	Died (*n *= 21)	***P***
**Male/female** **(n)**	52/42	42/31	10/11	n.s.
**Age** **(years)**	2.46	2.93	1.69	n.s.
**PC treatment**				
**PC total dose** **(IU/kg)**	258.8 (127.6-410.9)	277.8 (132.4-444.4)	153.8 (126.0-375.0)	n.s.
**PC daily dose** **(IU/kg/d)**	100 (73.4-136.6)	100 (78.74-133.3)	81.08 (71.09-153.8)	n.s.
**PC therapy duration (d)**	2 (1-4)	3 (2-4)	2 (1-4)	n.s.
**Bolus/** **Bolus + cont. inf**.	78/16	61/12	17/4	n.s.
**Haematological paramenters prior to PC treatment**				
**WBC** **(pl)**	10.4 (5.0-17.02)	11.3 (5.3-18.7)	7.21 (4.6-10.75)	n.s (0.062)
**CRP** **(mg/dl)**	10.48 (5.65-16.47)	11.6 (7.81-17.31)	5.9 (1.99-8.92)	0.0018
**Platelets** **(G/l)**	110 (66-183)	116 (74-182)	78 (52-178)	n.s.
**PT** **(%)**	41 (32-54)	44 (33-56)	31 (22-36)	< 0.001
**aPTT** **(sec.)**	59 (43-91)	52 (39-71)	108 (81-160)	< 0.001
**Fibrinogen** **(mg/dl)**	270 (174-440)	347 (217-503)	129 (82-202)	< 0.001
**D-Dimers** **(mg/l)**	2.38 (0.93-8.99)	2.13 (0.89-8.62)	6.40 (1.08-12.00)	n.s.
**AT** **(%)**	76 (57-87)	80 (60-88)	70 (46-80)	n.s.
**PC** **(%)**	27 (14-39)	30 (18-41)	10 (10-18)	< 0.05
**Haematological parameters during PC treatment**				
**WBC** **(pl)**	21.25 (12.75-27.28)	23.65 (13.65-28.75)	16.35 (7.7-20.90)	< 0.05
**CRP** **(mg/dl)**	14.70 (7.0-21.8)	15.91 (7.1-23.64)	9.36 (6.90-15.88)	n.s.
**Platelets** **(G/l)**	96 (57-130)	103 (65.5-136.5)	61 (30-80.75)	< 0.01
**PT** **(%)**	69 (48.5-87)	77.8 (55-91)	45.5 (37.75-55.75)	< 0.01
**aPTT** **(sec.)**	43 (33-52.75)	41 (33-47)	61 (47.5-88.4)	< 0.01
**Fibrinogen** **(mg/dl)**	558.5 (342-747.2)	600.5 (418-766)	214.5 (184-299.2)	< 0.01
**D-Dimers** **(mg/l)**	1.95 (0.8-6.16)	1.6 (0.68-5.96)	2.93 (1.14-6.4)	n.s.
**AT** **(%)**	87 (68.5-102.2)	87 (68-101.5)	78 (70-102)	n.s.
**PC** **(%)**	71 (53.5-108.4)	79 (54.7-106.8)	68.5 (33.75-108.5)	n.s.

Data on patient characteristics, outcome and laboratory findings. Shown as median and inter-quartile range (range between first and third quartile). Note that not all laboratory parameters could be obtained in all patients at each time-point.

aPTT, activated partial thromboplastin time; AT, antithrombin; CRP, C reactive protein; n.s., not significant; PC, protein C; PT, prothrombin time; WBC, white blood cell count.

The time interval between admission and the start of PC substitution was significantly longer (median 8.6 vs. 4 hours, *P *= 0.03) in patients who died compared with those who survived, and also longer in patients who had amputations and/or died compared with those who fully recovered (median 9.25 vs. 4 hours, *P *= 0.016).

### Inflammatory response

C-reactive protein (CRP) levels were significantly lower at admission in non-survivors compared with survivors (Median: 5.9 vs. 11.6 mg/dl, *P *= 0.002). White blood cell counts were also lower on admission and during treatment in the non-survivors, but this trend did not reach statistical significance (Table 1).

### Coagulopathy

Non-survivors showed a significantly higher prevalence than survivors of coagulopathy with prolonged activated partial thromboplastin time (aPTT) at admission (median: 108 vs. 52 seconds, *P *< 0.0001). A significantly higher rate of coagulopathy was still documented with PC treatment in the non-survivors (Table 1). The platelet count did not differ significantly at admission between survivors and non-survivors; however, during treatment, survivors showed significantly higher platelet counts than patients who died (median: 103 vs. 61 G/l, *P *= 0.0047).

### Fresh frozen plasma

Of the 94 patients, 71 received fresh frozen plasma (FFP): 45% received one FFP transfusion, 30% two and 25% received three or more FFP transfusions. The total amount of FFP given was 33.3 ml/kg in patients being transfused, on day one a median of 20 ml/kg, on day two a median of 22 ml/kg and on day three a median of 13 ml/kg. There was no difference in survival between those patients transfused compared with those who did not receive FFP (*P *= 0.345). However, there was a trend towards a better survival in those patients receiving high volume (≥ 25 ml/kg/d) FFP on day one compared with those who received less or no FFP (*P *= 0.069).

### Length of stay and mechanical ventilation

Length of stay on the ICU was a median of eight days (1 to 95 days) in the whole group, a median of two days for non-survivors and a median of nine days for survivors (*P *< 0.0001). The duration of mechanical ventilation was a median of 6.5 days for those who survived. Survivors were discharged out of hospital after a median of 18 days, and non-survivors had a median hospital stay of 2 days.

### Adverse events and hemorrhage

Four adverse events were reported in three patients. None was classified as severe by the treating physician (two events of hemorrhage from nose and/or throat, one event of pleural effusion, one event of transient increase in body temperature). The first patient with an adverse event developed bleeding from the nose six hours after receiving 66 IU/kg PC. The bleeding stopped spontaneously after one hour and the patient received the next scheduled dose two hours after the onset of the event without further complications. The treating physician classified the severity as moderate and causative relation to PC treatment as unknown. The second patient was a severely coagulopathic child, who developed hemorrhage from the throat and nose immediately after a difficult endo-tracheal intubation approximately 13 hours after receiving 83 IU/kg PC. The patient received the next dose as scheduled four hours after the event without further complications. The same patient developed a pleural effusion (serious with later blood staining) more that 24 hours after the PC therapy was ceased. The treating physician classified the severity of both events as moderate, a causative relation to PC treatment as unlikely. A third patient showed a transient (10 minutes) and minor increase in body temperature (from 39.0°C to 39.5°C) shortly after receiving 100 IU/kg PC. No treatment was required; the treating physician classified the severity as mild.

## Discussion

Here we report what is to date the largest series of pediatric patients with PF, as a consequence of acute PC pathway failure in sepsis, treated with PC concentrate. In the majority of patients, the underlying infection was identified as meningococcemia. PC substitution was well tolerated, safe and the need for amputations and skin grafts with 5.3% and 9.6%, respectively, both markedly lower than previously reported in children with PF.

PF, if associated with septic shock, is a devastating disease carrying a high mortality and significant morbidity. Although improvement in health care delivery for these very sick patients has dramatically improved survival in recent years [11,12], permanent disability as a result of amputation of limbs or digits, extensive scarring, or neurological injury remains problematic. Gurgey and colleagues reported a series of 16 children; nine (69%) of the 13 children aged 4 years or younger and one of the older children (age range 9 - 12 years) (33%) required amputation [13]. A case series by Wheeler and colleagues on 21 patients reported amputations in nine (43%) patients [14]. Recent data from Rotterdam reports amputations in 8%, skin grafts in 16% and skin scarring in 48% of survivors of meningococcal disease with PF, combined with orthopedic sequelae in 14% [15]. These patients did not receive PC. In five patients treated in a burn center including therapy with activated PC, an impressive 100% survival was achieved; however, amputations were still needed in two of the five children (40%) [16].

We and others have previously described replacement therapy with human, non-activated PC in patients with meningococcemia in case reports and smaller case series [3,4,6,17-19]. Other studies have explored the use of activated PC in patients with PF. Vincent and colleagues published a *post-hoc *analysis of recent studies using activated PC in adult and pediatric patients with severe sepsis, presenting with PF, meningitis or meningococcal disease [5]. The authors identified 119 pediatric patients suitable for the analysis, 87 of them with PF. In that group, which had a comparable incidence of coagulopathy and low PC plasma levels, but a slightly more severe thrombocytopenia compared with the group reported here (85; 42 to 122 vs. 110; 66 to 183 G/l median; first to third quartile), serious bleeding events were noticed in two patients during the infusion and in six patients over a 28-day period. Fourteen-day mortality was 9.4%, which is probably lower than the 22.3% in-hospital mortality in the group reported here. It is difficult to compare the illness severity of these two groups, because Vincent and colleagues did not report the rates for amputation or skin grafts. Another study, investigating the use of activated PC in children with severe sepsis, which was terminated prematurely due to lack of efficacy, showed an increased rate of hemorrhage in the activated PC compared with the placebo group, especially in children younger than 60 days [20].

In contrast, significant bleeding complications were not seen in our large group of pediatric patients and have also not been associated with the use of human, non-activated PC concentrate in adult studies so far.

As published previously, this study confirmed low plasma PC levels to be associated with negative outcome [21]. In fact, even a difference as small as 1% in PC plasma activity at admission changed the odds of survival significantly. For a number of medical conditions, minimizing the amount of time from patient presentation to initiation of treatment represents an important consideration in the improvement of treatment outcomes. Analysis of a large, hospital-level database suggested that earlier treatment with activated PC is associated with a lower in-hospital mortality in patients (*n *= 1179) with severe sepsis [22]. Another retrospective study, analyzing adults with severe sepsis and APC treatment found an increase in mortality from 33% if treated on the day of diagnosis to 40% if treatment was delayed by one day and to 52% if treatment was delayed by two days or more [23]. In our analysis, the interval between admission to ICU and the commencement of PC replacement therapy was significantly longer in patients who died compared with those who survived, which may point towards the benefits of early therapy in this rapidly progressive condition.

During PC therapy, coagulatory and inflammatory markers may be important prognostic markers. The prevalence of ongoing coagulopathy, as reflected in a significantly more abnormal aPTT and prothrombin time, and in significantly lower fibrinogen levels at admission and during therapy, was higher in non-survivors compared with survivors. During therapy, but not at admission, platelets were found to be significantly lower in patients who did not survive, also pointing towards ongoing coagulopathy as a negative prognostic marker. Interestingly, although non-survivors showed lower PC plasma levels and higher incidence of coagulopathy, the inflammatory response in terms of CRP levels and leukocytosis was less marked in the non-survivors, which could imply a degree of immuno-paralysis in those patients.

Being a retrospective multi-center analysis, the limitations of this study are obvious: the lack of a control group and a prospective design makes it impossible to comment of the effects of PC on survival. The patients were treated in many different centers with a consequently large potential for intra-observer variability. On the other hand, the fact that even with so many different centers and protocols involved, the safety profile was still favorable in this unselected high-risk population is very encouraging.

## Conclusions

This study shows encouragingly low rates of amputations and skin grafts in a large group of pediatric patients with PF, combined with improvement or resolution of PF in most of the patients across all pediatric age groups, with no significant adverse side effects. Our study supports the biological rationale of human non-activated PC concentrate as a treatment for severe acquired PC deficiency, presenting with PF. Future studies investigating the effect of human, non-activated PC concentrate should focus on the potential benefit of early PC therapy in PF, and amputations and skin grafts as important outcome measures in this condition, which, despite recent advances in mortality control, still carries a high risk of disabling long-term morbidity.

## Key messages

• Low plasma levels of PC are negatively correlated with survival in patients with PF in the context of meningococcemia.

• In this group of pediatric patients, substitution with human, non-activated PC concentrate resulted in an improvement of PF in the majority of patients, without causing significant bleeding.

• The need for amputations and skin grafts was low compared with historical controls, but there was no obvious effect on mortality.

• Future studies investigating the effect of human, non-activated PC concentrate should focus on early PC therapy in PF, with amputations and skin grafts as important outcome parameters.

## Abbreviations

aPTT: activated partial thromboplastin time; CI: confidence interval; CRP: C reactive protein; FFP: fresh frozen plasma; PC: protein C; PF: purpura fulminans.

## Competing interests

AV was a member of a Baxter advisory board and received as such an honorarium. At the time the study was performed, RS and BE were employees of Baxter Deutschland GmbH, Heidelberg, Germany. All authors had full and unrestricted access to the dataset. Baxter had no influence on the data selection, interpretation or publication.

## Authors' contributions

AV was involved in study design, data analysis and interpretation, and writing of the manuscript. DF, MS and FW were involved in data analysis and interpretation, and writing of the manuscript. WK was involved in study design and data analysis. BE was involved in study design and data collection. UM was involved in data management, statistical analysis and data interpretation. RS was involved in study design, data analysis, and writing of the manuscript. All authors read and approved the final manuscript.
